# Effects of endothelial defects and venous interposition grafts on the acute incidence of thrombus formation within microvascular procedures

**DOI:** 10.1038/s41598-021-88324-2

**Published:** 2021-04-22

**Authors:** Lucas M. Ritschl, Marie-Kristin Hofmann, Constantin T. Wolff, Leonard H. Schmidt, Klaus-Dietrich Wolff, Andreas M. Fichter, Thomas Mücke

**Affiliations:** 1 Department of Oral and Maxillofacial Surgery, School of Medicine, Klinikum Rechts Der Isar, Technical University of Munich, Ismaninger Str. 22, 81675 Munich, Germany; 2 Department of Oral and Maxillofacial Surgery, Helios Kliniken Rhein-Ruhr, Krefeld-Uerdingen, Germany

**Keywords:** Surgical oncology, Cardiovascular diseases, Experimental models of disease

## Abstract

Endothelial defects (ED) and the usage of interposition vein grafts (IVG) are known risk factors for free flap failure. This experimental study aimed to compare both situations of thrombus formation and fluorescence angiographic behavior. Indocyanine green videoangiography (ICGVA) with the FLOW 800 tool was systematically performed in groups I = ED, II = IVG, and III = ED and IVG (each *n* = 11). ICGVA was able to detect thrombosis in five animals and safely ruled it out in 26 with two false-positive cases (sensitivity, specificity, and positive and negative predictive values were 100%, 90%, 62%, and 100%, respectively). The difference between visually and ICGVA-assisted ED measurements was significant (*p* = 0.04). The areas of thrombosis showed no significant difference. Moreover, ICGVA detected a decrease of all parameters at the ED area and/or within the IVG section in all groups. The presence of an endothelial defect had a higher impact on thrombus formation than the IVG usage. ICGVA is qualitatively able to detect endothelial defects and clinically evident thrombosis. However, the quantitative values are not yet attributable to one of the clinical scenarios that may jeopardize free flap transfer.

## Introduction

Microvascular techniques have been established and developed within different surgical fields in the last decades. Free microvascular tissue transfer represents the gold standard for complex reconstructions in the head and neck area following ablative tumor surgery, excessive trauma, or other indications (e.g., because of side effects such as medication-related osteonecrosis of the jaw or osteoradionecrosis) in most specialized centers^1–3^.

On the one hand, this patient cohort is challenging because of multiple organic or vascular comorbidities with dissolved and altered vascular architecture resulting from arteriosclerosis, radiation therapy, or previous surgical interventions^4–7^. These patients need to be handled with a distinct approach in the pre-, intra-, and postoperative settings. Missed salvage operations of compromised free flaps, especially in this high-risk group, may result in increased rates of flap necrosis and consecutive prolonged hospital stay and costs^8–10^.

On the other hand, thrombosis still represents one of the most common reasons for flap loss despite the progress in microvascular surgery on the refinement of surgical techniques, instruments, and different monitoring devices. Borderline microsurgical situations, such as the irradiated and vessel depleted neck, are often associated with a hypercoagulable vascular state or may demand the use of an interposition vein graft (IVG), which is described by many authors as a high-risk factor for thrombosis^3,11,12^.

Previous studies revealed that the majority of thrombotic events appear within the first hours after performing an anastomosis^1^. Therefore, a high level of interest exists in an early, reproducible, and objective assessment of patency and blood flow^13^. However, indocyanine green (ICG), an injectable dye that binds to plasma proteins and remains intravascular, has been introduced to free flap surgery in the last two decades^14,15^. The intraoperative use of ICG videoangiography (ICGVA) in combination has increased, allowing quantitative and qualitative evaluation of blood flow and the detection of endogenous (e.g., endothelial defects) and exogenous (e.g., vascular compression due to hematoma) complications^13,16–21^.

This study aims to critically evaluate the diagnostic accuracy of ICGVA combined with the FLOW 800 tool in detecting microvascular thrombosis in three different procoagulant situations in a sensitive rat model^22^ by creating different potentially thrombogenic microvascular situations. Furthermore, this study also aims to compare thrombosis rates between endothelial defects and IVGs in an attempt to determine which combination creates a higher risk of thrombosis.

## Results

This study operated on 33 rats, and the infrarenal aorta had a median diameter of 1.72 mm (1.11–2.53). Moreover, no side effects attributable to ICG application were observed.

### Detection and determination of endothelial defect and thrombosis (Tables 1, 2)

**Table 1:** Cross table for ICGVA-assisted thrombus detection.

	Clinical/visual assessment		
	Positive	Negative	Total
ICGVA-assisted assessment			
Positive	5	3	8
Negative	0	25	25
Total	5	28	33

**Table 2:** Descriptive results of clinical ED area and thrombosis in visual and ICGVA analysis using NIH Image software (ImageJ 1.41o, National Institutes of Health, Bethesda, MD, USA).

Group	Visual area of thrombosis (mm^2^)	ICGVA area of thrombosis (mm^2^)	*p* value	Visual area of ED (mm^2^)	ICGVA area of ED (mm^2^)	*p* value
I	0.56 (0.36–1.06)	0.96 (0.42–1.14)	0.11	0.72 (0.25–2.30)	0.65 (0.35–1.59)	0.93
II	0.44	0.34		–	–	
III	–	0.36 (0.15–0.56)		0.19 (0.02–0.30)	0.30 (0.05–0.74)	< 0.01
Total	0.50 (0.36–1.06)	0.69 (0.15–1.14)	0.47^a^	0.26 (0.02–2.30)	0.39 (0.05–1.59)	0.04^a^

*ICGVA* Indocyanine green videoangiography, *ED* Endothelial defect, *group I* Endothelial defect, *group II* Interposition vein graft (IVG), *group III* Endothelial defect with interposition vein graft.

^a^Wilcoxon test.

A significant difference (*p* = 0.05) was noted between groups I and III in terms of the probability of thrombosis with an increased risk of thrombosis after endothelial defect only in contrast to the combined intervention. Further comparisons of the defect group (I) with the IVG alone group (group II) and between groups II and III showed no significant differences in terms of thrombus formation (*p* = 0.14 and *p* = 0.32, respectively).

Changes in ICGVA fluorescence behavior identified four, one, and two thromboses in groups I, II, and III, respectively. Visually (with a tenfold magnification), which served as reference, thrombus formation was however detected in four and one rats in groups I and II, respectively. However, no clinical evidence of thrombosis in any vessel was noted in group III. Furthermore, ICGVA was able to reliably detect thrombosis in five animals and safely rule it out in 26 animals, with two false-positive cases. This resulted in sensitivity, specificity, and positive and negative predictive values of 100%, 90%, 62%, and 100%, respectively (Table 1).

The area of endothelial defect (visually and ICGVA-assisted) and the two-dimensional measurement of the extent of thrombosis (visually and ICGVA-assisted) are displayed in Table 2. Overall, a significant difference was noted between visual and ICGVA-assisted measurements of ED (*p* = 0.04), even though the measurements in group I were comparable (0.72 mm^2^ [0.25–2.30] vs. 065 mm^2^ [0.35–1.59]; *p* = 0.93). The areas of thrombosis showed no significant difference, either overall (*p* = 0.47) or in group I (*p* = 0.11).

### Impact of the endothelial defect and thrombus formation on ICGVA (Tables 3, 4)

**Table 3:** Absolute values (arbitrary units, AU) and descriptive statistics of ICGVA for the three experimental groups: endothelial defect (ED group I), interposition vein graft (IVG, group II), and ED with IVG (group III).

ROI no	Parameter	Group		
		I	II	III
#1	First maximum	345.07 (181.33–575.83)	378.28 (193.15–661.36)	566.49 (180.12–758.49)
	Maximal increase	0.59 (0.27–0.75)	0.64 (0.18–1.84)	0.69 (0.28–3.79)
	Maximal decrease	− 0.10 (− 0.38–(− 0.05))	− 0.28 (− 2.71–(0.03))	− 0.19 (− 0.57–(− 0.08))
	AUC^ICG^	327,850.14 (134,045.89–483,015.33)	404,530.41 (147,937.59–656,325.44)	498,672.58 (202,527.95–586,814.66)
#2	First maximum	140.63 (102.39–224.89)*	–	410.88 (124.67–594.93)*^,&^
	Maximal increase	0.20 (0.12–0.75)*	–	0.52 (0.19–2.69)*^,&^
	Maximal decrease	− 0.10 (− 0.89–(0.04))	–	− 0.16 (− 0.54–(− 3.61))
	AUC^ICG^	158,315.35 (78,304.80–279,790.88)*	–	295,815.71 (152,913.84–398,368.67)*^,&^
#3	First maximum	–	281.25 (108.75–552.15)^§^	394.55 (135.04–549.57)^§^
	Maximal increase	–	0.53 (0.15–1.74)	0.49 (0.22–2.46)^§^
	Maximal decrease	–	− 0.22 (− 1.56–(− 0.11))	− 0.14 (− 0.46–(− 0.05))
	AUC^ICG^	–	296,593.93 (124,613.19–363,385.51)^§^	340,056.17 (149,678.67–431,823.48)^§^
#4	First maximum	318.66 (209.17–536.32)	333.03 (118.44–644.13)	508.60 (187.70–626.00)^#,?^
	Maximal increase	0.53 (0.33–0.98)	0.58 (0.16–1.05)	0.73 (0.16–2.63)
	Maximal decrease	− 0.14 (− 0.59–(− 0.06))	− 0.36 (− 1.22–(− 0.09))	− 0.13 (− 0.69–(− 0.07))
	AUC^ICG^	327,119.88 (141,131.65–441,150.91)	368,974.45 (185,822.91–464,508.97)^#^	380,680.60 (180,753.86–520,915.12)^#^

*I* defect, *II* interposition vein graft, *III* defect and interposition vein graft, *ROI* region of interest, *AUC*^*ICG*^ area under the curve.

Median (range).

Wilcoxon test: significant (*p* < 0.05) change in ROI 1 vs. 2, 3, and 4 (*,^§^, ^#^) within groups I–III and compared values.

Mann–Whitney U test: significant (*p* < 0.05) change between groups I vs. II, I vs. III, and II vs. III (^%^, ^&^, ^?^) for the compared values.

**Table 4:** Differences of fluorescence behavior (AU) in ICGVA between the defined regions of interest (ROI, 1–4) for the three experimental groups: endothelial defect (ED group I), interposition vein graft (IVG, group II), and ED with IVG (group III).

	Parameter	Groups		
		I	II	III
∆ ROI 1 and 2	First maximum	122.38 (9.99–377.92)	–	200.30 (0.00–329.93)
	Maximal increase	0.23 (− 0.02–0.49)	–	0.10 (0.02–1.11)
	Maximal decrease	0.02 (0.20–0.73)	–	0.01 (− 0.20–3.45)
	AUC^ICG^	169,534.79 (− 2062.07–344,419.32)	–	119,513.21 (28,090.56–277,780.77)
∆ ROI 1 and 3	First maximum	–	76.44 (− 0.91–276.86)	185.97 (23.98–290.91)
	Maximal increase	–	0.09 (− 0.33–0.46)	0.09 (− 0.06–1.34)
	Maximal decrease	–	0.00 (− 1.87–0.20)	0.02 (− 0.17–0.07)
	AUC^ICG^	–	82,172.10 (− 1,286.26–359,731.52)	154,991.18 (52,849.27–201,659.19)
∆ ROI 1 and 4	First maximum	39.51 (− 174.00–107.49)	− 15.54 (− 171.90–172.06)	90.07 (− 165.91–271.81)
	Maximal increase	0.02 (− 0.37–0.18)	− 0.05 (− 0.40–0.83)	0.08 (− 0.60–1.17)
	Maximal decrease	0.03 (− 0.04–0.21)	0.04 (− 2.30–0.23)	0.01 (− 0.16–0.19)
	AUC^ICG^	35,304.97 (− 11,221.14–110,432.71)	− 3,990.70 (− 113,622.70–314,222.50)	119,735.07 (− 82,006.72–174,410.91)

*I* defect, *II* interposition vein graft, *III* defect and interposition vein graft, *ROI* region of interest, *AUC*^*ICG*^ area under the curve.

Median (range).

Wilcoxon test: significant (*p* < 0.05) change in ROI 1 vs. 2, 3, and 4 (*,^§^, ^#^) within groups I–III and compared values.

Mann–Whitney U test: significant (*p* < 0.05) change between groups I vs. II, I vs. III, and II vs. III (^%^, ^&^, ^?^) for the compared values.

**Figure 1:**
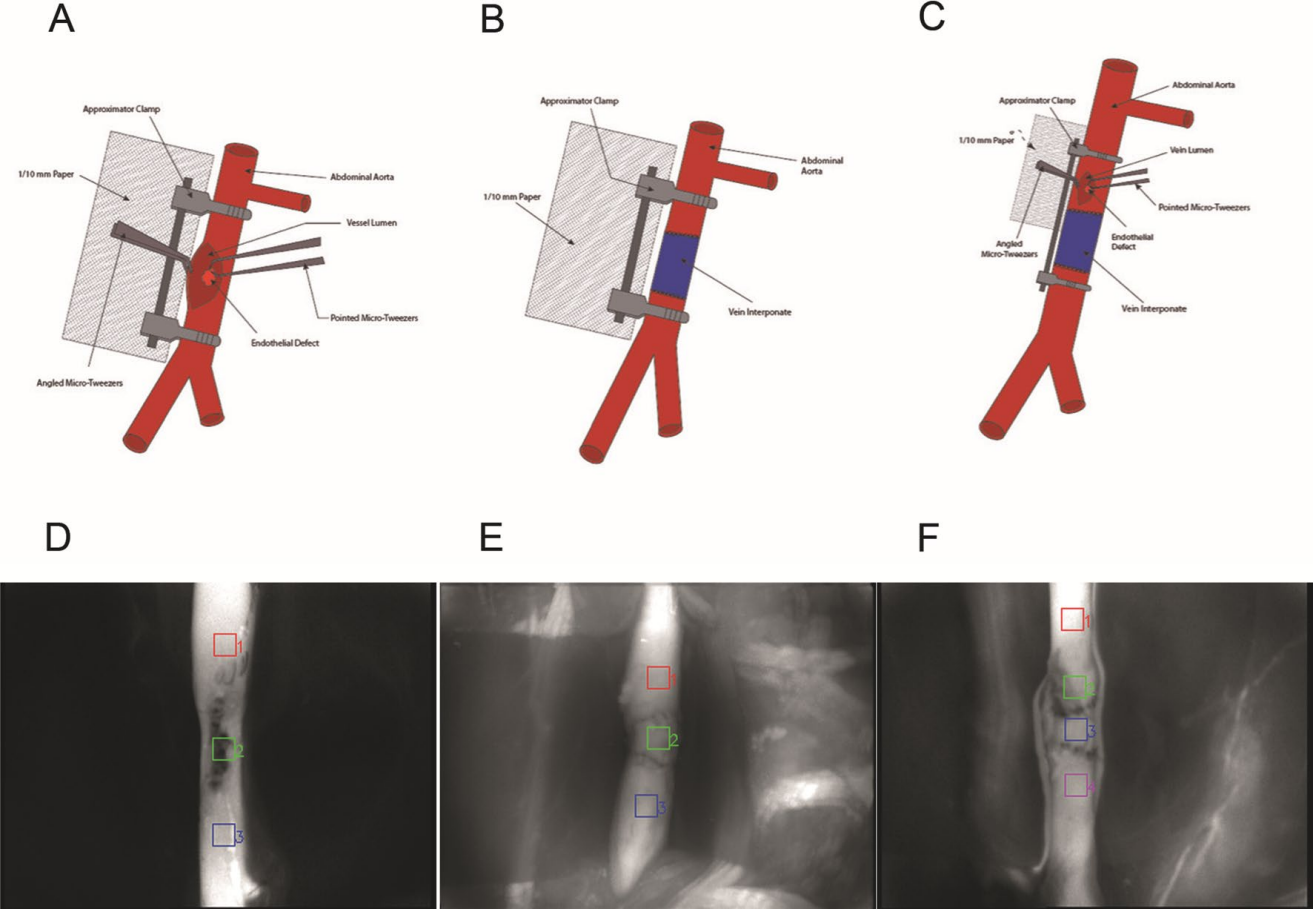
Three experimental groups. (**A**) and (**D**) endothelial defect (ED, group I); (**B**) and (**E**) interposition vein graft (IVG, group II); and (**C**) and (**F**) ED with IVG (group III) with corresponding regions of interest (ROI, 1–4).

All four defined parameters (first maximum, maximal increase and decrease, and area under the curve (AUC^ICG^)) of the detected ICG fluorescence curve were calculated for each region of interest (ROI) to compare them within and between the groups (Table 3). Additionally, the differences between ROIs 1 and 2, 1 and 3, and 1 and 4 were calculated and are displayed in Table 4. All three groups (I–III) showed a decrease in all parameters within the ROIs at the defect area and/or within the area of the IVG (Table 3 and Fig. 1). The value of the first maximum significantly changed between ROI 1 vs. 2 in group I (*p* < 0.01); between ROI 1 vs. 3 in group II (*p* < 0.01); and between ROI 1 vs. 2 (*p* < 0.01), ROI 1 vs. 3 (*p* < 0.01), and ROI 1 vs. 4 (*p* = 0.05) in group III. The maximal increase between the following ROIs was significantly different: ROI 1 vs. 2 (*p* < 0.01) in group I and between ROI 1 vs. 2 (*p* < 0.01) and ROI 1 vs. 3 (*p* = 0.01) in group III. The values of maximal decrease showed no significant changes within the different ROIs for each group. The values of AUC^ICG^ significantly changed between ROI 1 vs. 2 in group I (*p* < 0.01), between ROI 1 vs. 3 in group II (*p* < 0.01), and in group III between ROI 1 vs. 2 (*p* < 0.01), ROI 1 vs. 3 (*p* < 0.01), and ROI 1 vs. 4 (*p* = 0.01).

The comparison of registered corresponding ICG values of each ROI (2, 3, and 4) between groups I–III showed a significant difference for first maximum (*p* < 0.01), maximal increase (*p* = 0.02), and AUC^ICG^ (*p* < 0.01) within the corresponding ROI 2 between groups I and III. A comparison of registered ICG values within corresponding ROI 3 between groups II and III revealed no significant differences. A comparison of the ICG values within corresponding ROI 4 between groups I vs. II and II vs. III revealed no significant differences. Only the first maximum was significantly different between groups I and III (*p* = 0.03) within ROI 4.

### Uni- and multivariate regression analyses (Table 5)

**Table 5:** Univariate regression analysis of incidence of thrombosis, visual area of thrombosis, and visual area of endothelial defect of the analyzed ICGVA parameters.

ROI 1	First maximum (95% CI)	Maximal increase (95% CI)	Maximal decrease (95% CI)	AUC^ICG^ (95% CI)
Incidence of thrombosis	0.35 (− 267.17–97.23)	0.19 (− 1.05–0.21)	0.28 (− 0.23–0.77)	0.68 (− 101,600.43–698,051.60)
Area of thrombosis	0.34 (− 937.43–463.27)	0.96 (− 0.93–0.96)	0.66 (− 0.21–0.15)	0.72 (− 653,469.09–510,601.89)
Area of ED	0.01 (− 323.11–(− 44.23))	0.25 (− 0.94–0.26)	0.20 (− 0.04–0.17)	0.92 (− 517,867.41–191,121.70)

ROI 2	First maximum (95% CI)	Maximal increase (95% CI)	Maximal decrease (95% CI)	AUC^ICG^ (95% CI)
Incidence of thrombosis	0.16 (− 317.83–56.82)	0.30 (− 0.96–0.31)	0.52 (− 1.29–0.68)	0.20 (− 190,852.74–43,431.62)
Area of thrombosis	0.60 (− 310.80–414.97)	0.38 (− 0.54–0.93)	0.69 (− 2.91–2.34)	0.12 (− 99,464.44)–422,500.89)
Area of ED	0.04 (− 260.72–(− 8.12))	0.19 (− 0.74–0.16)	0.55 (− 0.91–0.49)	0.03 (− 165,791.55–10,858.94)

ROI 3	First maximum (95% CI)	Maximal increase (95% CI)	Maximal decrease (95% CI)	AUC^ICG^ (95% CI)
Incidence of thrombosis	0.67 (− 240.35–366.56)	0.35 (− 1.71–0.64)	0.59 (− 0.56–0.95)	0.87 (− 169,413.58–200,025.36)
Area of thrombosis	–	–	–	–
Area of ED	0.64 (− 920.09–1426.71)	0.55 (− 3.93–6.91)	0.25 (− 1.40–0.41)	0.59 (− 524,308.72–869,927.95)

ROI 4	First maximum (95% CI)	Maximal increase (95% CI)	Maximal decrease (95% CI)	AUC^ICG^ (95% CI)
Incidence of thrombosis	0.91 (− 136.73–153.81)	0.42 (− 0.64–0.28)	0.15 (− 0.07–0.41)	0.72 (− 78,677.48–112,855.86)
Area of thrombosis	0.49 (− 583.95–357.12)	0.71 (− 1.15–1.49)	0.26 (− 0.10–0.26)	0.55 (− 311,891.11–479,577.77)
Area of ED	0.11 (− 183.62–19.72)	0.32 (− 0.64–0.21)	0.63 (− 0.11–0.18)	0.19 (132,104.38–29,150.71)

*95% CI* 95% Confidence interval, *ROI* Region of interest, *AUC*^*ICG*^ Area under the curve.

Univariate regression analysis for thrombosis formation and visual (reference) detection showed a significant association with the size of endothelial defect (mm^2^; *p* < 0.01; 95% confidence interval (CI), 0.42–0.78) and usage of an IVG (*p* = 0.02; 95% CI, − 0.57–(− 0.07)). Multivariate regression analysis only showed a significant association with the size of endothelial defect (mm^2^; *p* < 0.01; 95% CI, 0.40–0.89).

## Discussion

Technically meticulous flap raising, correct and gentle handling of vessels in microvascular preparation and technically flawless microsurgical anastomosis are essential and mandatory requirements in reconstructive microsurgery. Nevertheless, flap failure rates for head and neck reconstruction have ranged between 0 and 7.7% in the literature over the decades^23,24^. These generally good results also include cases that were surgically reexplored (*set back*) in the operation room because of an imminent loss of free flap^25^. However, success can only be assumed if the salvage operation was performed within transplant-specific congestion or ischemic time interval in the case of a successful revision of the anastomosis or elimination of the decisive cause^26,27^. Although the sensitivity and specificity of ICGVA have already been investigated in a few clinical and animal studies, the simulation of complicated microsurgical situations requiring an IVG has not yet been investigated. A more realistic vascular analog model to a demanding clinical situation was created and evaluated by setting endothelial defects and/or using an IVG.

The first 72 postoperative hours remain the main interval for free flap loss^1,28^. Sweeny et al. and others described that flap loss attributable to venous congestion occurs earlier than flap loss due to arterial insufficiency or infection^28^. Clinically, persisting arterial vasospasm, endothelial cell damage, or inadequate microsurgical sutures (e.g., intraluminal adventitia) subsequently lead to early thrombus formation and intraoperative occlusion. Thus, a clinical observation period of at least 45 min is recommended, during which, for example, reconstruction with the free flap can be performed^1^. Late free flap failure may have different reasons, which can be classified into surgical (including intra- and extravascular) and nonsurgical (including alcohol withdrawal) causes. Based on this knowledge, this study has chosen the acute observation period of 60 min similar to Mordick et al.^29^ to analyze the possibly altered perfusion changes after a reestablished blood flow after approximator clamp removal. An earlier timepoint would *only* reflect immediate changes. Scientifically, earlier and later timepoints than 60 min may be of interest as well. However, clinically relevant is the chosen timepoint because the greatest number of acute, anastomosis-related flap losses demark within this time.

Even though venous thrombosis and consecutive malperfusion occur more often than arterial malperfusion, this study chose to vein graft the arterial over the venous system. One reason was that interposition vein grafting in an artery may be associated with a higher impact on blood flow disturbances due to venous sacking and turbulent flow. Consequently, Zhang et al. reported comparable patency rates in arterial and venous vein grafted femoral vessels and in free groin flap in the rat^30,31^. Another reason for the choice of the arterial system was to analyze the ICGVA capacity and relevance of vessel alteration in a model which approximates peripheral arteriovenous vascular disease because this group of patients will also increase over time and will need to receive further attention in reconstructive microsurgery.

The early and reliable detection of an impending loss is of particular interest if a transplant failure does occur. Various instrumental techniques have been described for this purpose in addition to the clinical examination^32–34^. The results of our presented study put the detection one step before free flap failure and might help in the decision-making of the correct vessel choice. On the basis of our results, detection of endothelial defects and potential thrombosis after microvascular anastomosis is possible. This insight can increase the intraoperative safety, especially for less experienced colleagues. The results of this study placed the detection one step before free flap failure and may help in the decision-making of the correct vessel choice. Based on the results of this study, the detection of endothelial defects and potential thrombosis after the microvascular anastomosis is possible. This insight can increase intraoperative safety, especially for less-experienced colleagues.

Alongside the known Virchow’s triad—endothelial alteration/defect, blood flow alteration, and changes in the blood’s viscosity—other endo- and exogenic factors may contribute to thrombus formation. The results of this study suggest that endothelial defects may have a more harmful effect on the success of microvascular interventions than the use of interposition vein grafts. Wolff et al. also described that the endothelial defect configuration has an impact on thrombosis formation, whereby the vertical defects were associated with increased risk^35^. Moreover, Seo et al. histologically examined the anastomotic areas of five failed free flaps to determine the cause of failure. Consequently, thrombotic material and additional endothelial defects were detected in all anastomotic areas^36^. Furthermore, the endothelial defect size significantly correlated with thrombus formation in the uni- and multivariate regression analyses (Table 5). More thromboses were detected in group I compared with groups II and III. This fact puts the correct diagnosis of a (pre-)existing endothelial defect in a new and important light. Interestingly, ICGVA was able to achieve comparable results as the visual examination at tenfold magnification in the measurement of the defect size (Table 2). ICGVA was able to reliably detect a thrombus event (sensitivity and negative predictive value each 100%). However, an exact assessment of the presence of a thrombus can be generally difficult due to overlapping phenomena, e.g., vascular sutures. Furthermore, the exact delineation of a thrombus is sometimes difficult and depends on the subjective assessment of the surgeon although fluorescence reduction in thrombosed vessels could be visualized in this study. In difficult situations, it may therefore be useful not to rely solely on ICGVA but to use a combination of different methods such as Doppler or clinical inspection.

The fluorescence behavior showed a significant reduction of the first maximum, maximal increase, and AUC^ICG^ (Tables 3, 4). However, no significant correlation could be described in univariate regression analysis (Table 5). The reason for this may be the small number of animals as well as the low count of evident thrombus formations. This study failed to associate the incidence of thrombosis with a certain numerical or quantitative value in ICGVA, but this failure could also be a result of the lack of statistical power for this precise question.

Mücke et al. have previously analyzed the feasibility of ICGVA to detect immediate arterial thrombus formation. The authors observed that macroscopical analysis correlated well with ICGVA^22^. Thus, a histological examination was not included in this study. The results of the current study corroborate Mücke et al.’s observation that ICGVA is a valuable method to detect early thrombosis. Compared with other methods, ICGVA is user-friendly and offers real-time, radiation-free investigation without the need for a radiologist performing the diagnostics, allowing an independent examination. Despite the advantages, good tolerability, and the variable application possibilities of ICGVA, various disadvantages and limitations of the method exist. The vessel section of interest in ICGVA must be exposed to make fluorescence angiography possible. Vascular structures that are covered with blood clots or tissue, for example, cannot be adequately examined, which consecutively analyzes of buried flaps and hidden vessels impossible. Moreover, the dye is applied in an additive pattern in which the arterial and venous phases may overlap. Therefore, simultaneous assessments of multiple structures, which may require multiple applications of ICG are more difficult and prone to falsely increase fluorescence signal measurements. Blood leakage can also lead to increased fluorescence signals because the extravasated dye remains adjacent to the vessel. This may have led to somehow increased values in group III because most blood leakages were noted. The washout period of 10 min must be respected after each application, leading to a prolongation of the operation time as well as to artifacts and thus a more difficult assessment of the vascular situation. However, the problem associated with multiple administrations has not been an issue of this study because only one ICG bolus was given.

Generally, vein grafts are not often needed in the clinical setting and are only used in 1%–7% of free flap transfers in high-volume centers^37,38^ as a valuable and feasible method and exit strategy out of trouble to lengthen the pedicle and aiding the inset and shaping the flap^39^. Its association with increased flap loss is controversial^30,37,38,40,41^ and seems to be currently refuted because more reflected and critical clinical studies have been performed that have unprejudicedly worked on this question^37,39,41^. Nevertheless, several proposed mechanisms exist for the reported increased thrombotic rates associated with the use of vein grafts. These include multiple anastomoses, turbulent or altered flow dynamics, intrinsic thrombogenicity of the vein graft, and handling a flaccid vein graft promoting compression, twisting, and kinking^30,38,42,43^.

The usage of a vein graft was not associated with increased thrombosis formation in this animal study, where the vein graft was used as an IVG. Comparable good patency results were reported by Mordick et al., who compared reversed vs. nonreversed interposition vein grafts in the femoral artery in a rabbit model^29^. Generally, one could argue that the length of the IVG (10 mm) used in this study did not correspond to the relative length in most clinical settings, especially in lower extremity reconstructions^40^. The resulting blood flow changes within the IVG in the clinical setting are only approximately simulated in this study. However, Büchler and Buncke considered the IVG ratio to be the recipient vessel of 1:1 to 1:1.5 to be optimal to avoid blood flow disturbances and consecutive thrombosis^44^. This ratio was maintained in this study so that no vessel mismatch occurred. The average vessel diameter of the abdominal aorta was 1.72 mm and the known diameter of the femoral vein is 1.1 mm on average^45^.

Clinically, Cheng et al. could not conclude a causal cause of free flap failure because of the need for vein grafts, even though the failure rate was higher in the pooled vein-grafted group compared with the nonvein-grafted flap transfers in their literature review^46^. Like others, the distinct and unreflected perception—vein graft represents an independent risk factor for flap failure—was assumed as selection biased by the underlying circumstances that necessitated its use. Vein grafts, which are based on a preoperative plan because of pedicle lengthening due to traumatic or oncologic events or maintain and salvage venous outflow appear safer than vein grafts, which are a creative solution to an intraoperative dilemma^39,41^. Nelson et al. compared in this context the primary and secondary applied interposition vein grafts in a retrospective single-center study. They described that salvaged cases, where IVGs were utilized, had significantly higher failure rates compared with primary reconstructions. However, primary vein grafted reconstructions were reported to have a 95% overall flap success rate. Moreover, a 100% success rate was seen in preoperatively planned IVG^39^. Maricevich et al. reviewed 3,240 free flap cases wherein 241 IVG was used (7.4%) and described a free flap loss rate of 1.1% vs. 6.4%, which was statistically significant. The cause (anastomotic error, vein graft thrombogenicity, and extrinsic factors) for the increased flap loss rate in the vein-grafted group ultimately remained unknown. However, the vein-grafted group was significantly more often irradiated, previously operated, had multiple free flap operations in their analysis, which raises the question for selection bias because may be difficult to ascertain the exact cause of flap loss in many cases to definitively rule out any inherent effect of the overall vein graft. Additionally, the type of vein graft may also impact the incidence of take-back and success. Furthermore, Inbal et al. described more take-back and free flap failures in vein grafts requiring two microsurgical anastomotic suture lines like in the IVGs versus the one seen in transposition and arteriovenous loops^41^.

In summary, based on these studies as well as the current results, the use of an IVG itself can be assumed to not be associated with an increased risk of thrombosis or flap loss if all controllable iatrogenic factors are respected and the procedure is performed by an experienced microsurgeon even though the underlying indication of an IVG reflects the clinical and microsurgical complexity.

The nature of the study (animal) does not allow direct transfer to the clinical situation because it represents a maximal standardized situation in an animal model that will not approach the microsurgical complexity when IVGs are used. Nevertheless, this study tried to generate a more realistic vascular model analog to the demanding clinical situation that necessitates interposition vein grafting by setting endothelial defects and/or using an IVG. However, the rat model remains the gold standard experimental model for microvascular procedures because it offers comparable physiology, rheology, and coagulative behavior as the human being.

## Methods

### Ethical statement and animal welfare

The study conforms with current German regulations, guidelines for animal welfare, and the international principles of laboratory animal care. The study was carried out following the ARRIVE guidelines and the local government approved the animal experiments (Regierung Oberbayern; AZ.: 03-35-08). The animals were housed in filter-top cages under hygienic conditions according to the Federation of Laboratory Animal Science Associations (FELASA) guidelines. Water and standard rodent diet (Altromin; Altromin Spezialfutter GmbH & Co. KG; Lage, Germany) were provided ad libitum. All animals were sacrificed following a standardized protocol at the end of the measurements and observation time^47^.

### Workflow, model generation, and experimental groups

The anesthesia was induced with a weight-dependent intraperitoneal injection of ketamine–xylazine (1 and 0.25 mL/kg/weight, respectively). The anesthesia was then continued intravenously using femoral vein access after insertion of a microcatheter (Premicath; VYGON GmbH & Co. KG; Aachen, Germany). Maintenance dosage was a one-eighth dose of 10% ketamine when needed, as previously described in detail^17,48^.

**Figure 2:**
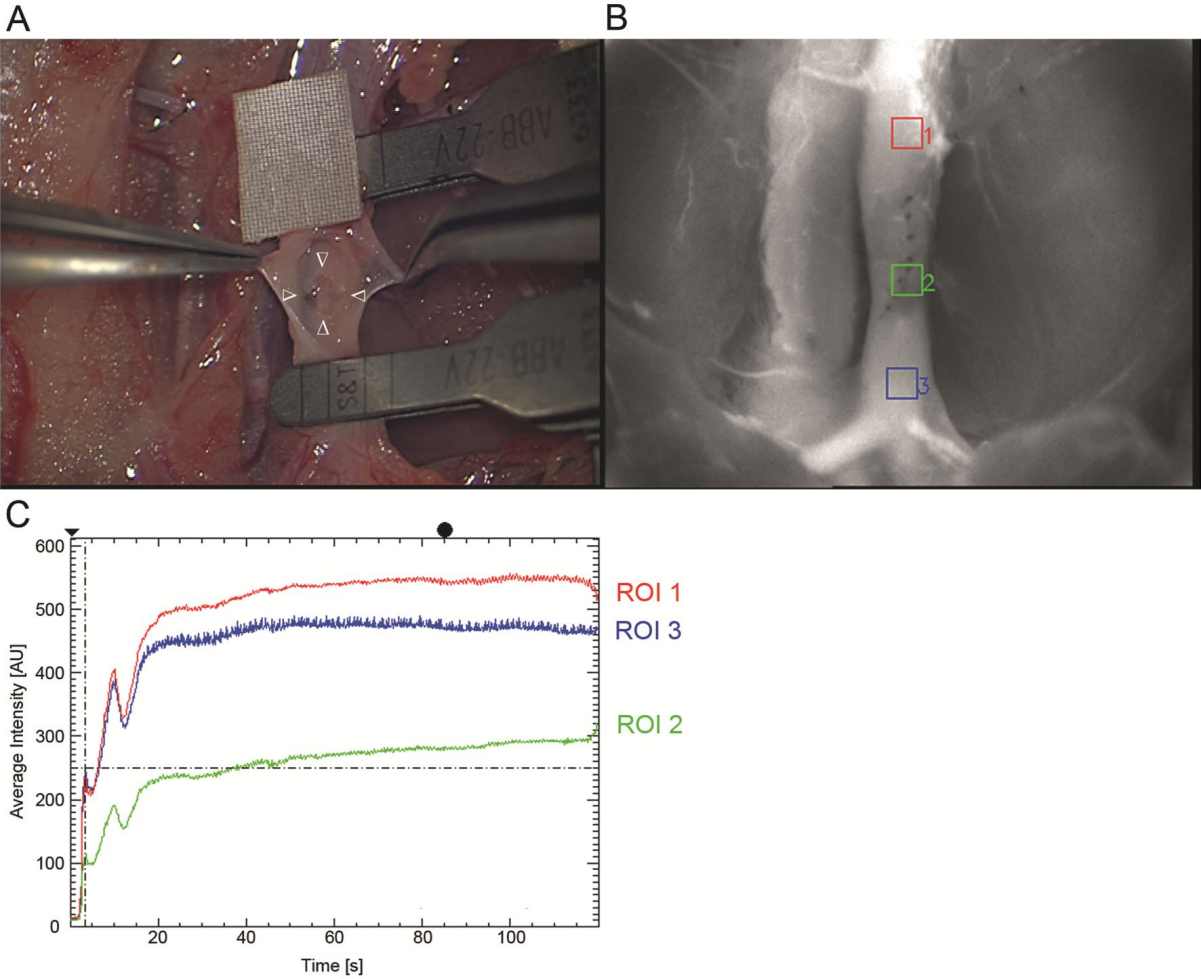
Endothelial defect (group I). (**A**) Prepared defect area (∆ indicating the borders of the defect), (**B**) three regions of interest (ROI, 1 = proximal, 2 = in, and 3 = distal to the ED), and (**C**)corresponding fluorescence intensity curve (in AU) for capturing time of 120 s.

The abdomen was shaved, and an approximately 5 cm midline laparotomy was performed. The prolapsing intestine was carefully retracted with moist swabs and atraumatic, blunt hooks. The retroperitoneal space was opened, the abdominal aorta was defined, the perivascular sheet was incised, and the infrarenal aorta was carefully freed from the surrounding tissue and separated from the vena cava inferior. All branching arterial vessels were ligated and cut. The preparation was randomized for the subsequent procedures according to three experimental groups (each *n* = 11): endothelial defect (ED, group I), interposition vein graft (IVG, group II), and ED with IVG (group III; Fig. 1). The EDs were generated with microforceps by removing the endothelial layer within a distinct area opposite to a longitudinal incision (Fig. 2A)^17^. A 1/10-mm paper was positioned adjacent to the defect on the aorta to enable an orientated and standardized defect size preparation. The femoral vein served as an IVG donor site. It was harvested with a standardized length of 10 mm distal of the inserted and fixed microcatheter. Before microsurgical grafting, the IVG was rinsed with heparin-diluted highly purified water and gently dilated with a dilatator (D-5a, S&T AG; Neuhausen, Switzerland).

After all group-related surgical procedures, microvascular anastomosis and longitudinal incision closure were performed with interrupted sutures using 10–0 Ethilon (Ethilon; Ethicon Division of Johnson & Johnson; Livingston, Scotland). The temporary Acland approximator (ABB-22 V, S&T AG; Neuhausen, Switzerland) was then removed and the blood flow was reestablished.

### Indocyanine green fluorescent videoangiography and analysis

Intraoperative ICGVA was previously performed as described in detail^16,17^ 60 min after blood flow reestablishment following the aforementioned surgical procedures. Indocyanine green dye (ICG-PULSION; Pulsion Medical System AG; Munich, Germany) was weight-dependently (0.03 mg/kg body weight) injected intravenously to assess the fluorescence behavior using the operating microscope type OPMI Pentero with an integrated near-infrared videoangiography detection system and the FLOW 800 tool (INFRARED 800; Carl Zeiss Meditec AG; Oberkochen, Germany)^49,50^. Fluorescence angiography was conducted at a fixed working distance of 300 mm and with a tenfold magnification. The ICG dye-specific emission was detected and color encoded concerning fluorescence intensity over time (AU)^13^. The immediate analysis of three or four regions of interest (ROI) was systematically positioned. The number of ROIs depends on the group. Group I had three ROIs (proximal, inside, and distal to the ED), group II had three ROIs (proximal, inside, and distal to the IVG), and group III had four ROIs (proximal to the ED and IVG, inside the ED, inside the IVG, and distal to the ED and IVG). The values of the first maximum, maximal increase to the first maximum, maximal decrease after first maximum, and the AUC^ICG^ of the resulting fluorescence curve over time were captured for 120 s with 25 images per second and analyzed within all ROIs (Fig. 2B,C).

### Analyses and correlation of incidence of thrombosis and defect area

The incidence of thrombosis was dichotomously analyzed. Visually, a macroscopic clot at a tenfold magnification and a spearing of the fluorescent signal in ICGVA were rated positively for thrombosis formation.

The defect size of the endothelium (in square millimeter) was planimetrically measured using NIH Image software (ImageJ 1.41o, National Institutes of Health; Bethesda, MD, USA) both for clinical macroscopic and ICGVA analyses^51,52^.

### Statistical analyses

Fisher’s exact test was used to determine the thrombosis probability of groups I–III. The Wilcoxon test was used to analyze the measurements of two related samples (development of ICG fluorescence behavior within groups I, II, or III). Moreover, the Mann–Whitney *U* test was used to compare the registered corresponding ICG (first maximum, maximal increase and decrease, and AUC^ICG^) values of each ROI (2, 3, and 4) between groups I–III. For the incidence of clinically proven thrombosis, uni- and multivariate regression analyses were performed with the clinically measured ED size and the IVG usage. *P* values are two-sided and subject to a global significance level of 0.05. The data were analyzed using the Statistical Package for the Social Sciences, version 23.0, for Windows software (IBM Corp; Armonk, NY, USA).

## Conclusions

The presence of endothelial defects had a higher impact on thrombus formation than the usage of an IVG in the abdominal aorta model of the rat. Moreover, indocyanine green videoangiography using the FLOW 800 tool is qualitatively able to detect endothelial defects and clinically evident thromboses. Significant quantitative values are not yet attributable to one of the clinical scenarios that may jeopardize successful free flap transfer. Thus, further research is needed to translate these observations into the clinical setting.

## Data Availability

All data generated or analyzed during this study are included in this published article.
